# Real role of β-blockers in regression of left ventricular mass in hypertension patients: Bayesian network meta-analysis

**DOI:** 10.1097/MD.0000000000006290

**Published:** 2017-03-10

**Authors:** FuWei Xing, Jialin Chen, BinLiang Zhao, Jingzhou Jiang, Anli Tang, Yili Chen

**Affiliations:** a Department of Cardiology, First Affiliated Hospital of Sun Yat-sen University; b Zhongshan School of Medicine, Sun Yat-sen University; c Nuclear Medicine Department, First Affiliated Hospital of Sun Yat-sen University, Guangzhou, Guangdong, People's Republic of China.

**Keywords:** anti-hypertensive drug, Bayesian network analysis, β-blockers, hypertension, left ventricular hypertrophy

## Abstract

**Background:**

Left ventricular hypertrophy (LVH) is commonly present in patients with hypertension (HT). According to the expert consensus document from American, angiotensin-converting enzyme inhibitor (ACEI) and angiotensin receptor blockers (ARBs) were recommended as 1st-line therapeutic drugs. However, none noticed the different efficacy between fat-soluble and selective β1-receptor blockers (FS-β-B) and other β-blockers on regression of LVH before. The aim of this analysis was to compare the efficacy of FS-β-B with the other 4 different classes of antihypertensive drugs (ACEI, ARBs, calcium channel blockers [CCBs], and diuretics) on regression of LVH.

**Methods:**

Relative trials were identified in the PubMed, Web of Science, OVID EBM Reviews and Cochrane databases, and the relevant papers were examined. We performed both traditional and Bayesian meta-analysis of randomized controlled trials (RCTs) about the regression of LVH. Sensitivity analysis and regression analysis were performed to explore possible sources of heterogeneity. Inconsistency analysis was performed to check whether the analysis of the trials in the network was indeed consistent.

**Results:**

A total of 41 RCTs involving 2566 patients with HT and LVH were included in this analysis. Bayesian network meta-analysis indicated no statistically significant differences between these groups: FS-β-B and ACEI (MD, −7.09; 95% CI, −14.99, 1.27); FS-β-B and ARB (MD, −2.66; 95% Cl, −12.02, 6.31). Although FS-β-B showed greater efficacy when compared with diuretic (MD, 13.04; 95% CI, 3.38, 22.59) or CCB (MD, 10.90; 95% CI, 1.98, 19.49). The probabilities of being among the most efficacious treatments were: FS-β-B (72%), ARB (27%), ACEI (0.01%), CCB (0.00%), and diuretic (0.00%).

**Conclusion:**

Evidence from our analysis reveals that FS-β-B have potential to become 1st-line therapeutic drugs in HT and LVH patients. However, the real efficacy of FS-β-B on regression of LVH should be confirmed by further large, high quality trials considering the limitation of the study number.

## Abstract 2

Supplemental Digital Content is available in the text

## 1 Introduction

Left ventricular hypertrophy (LVH) is commonly present in hypertensive (HT) patients. It could strongly predict cardiovascular mortality and morbidity,^[1–3]^ and was associated with increased incidence of atrial fibrillation, left ventricular dysfunction, and heart failure.^[4–6]^

There were several meta-analyses concerning the effect of 5 different classes of antihypertensive drugs on LVH.^[7–9]^ Although their conclusions had some differences, all of them agreed on a point that regression was worse with β-blockers and better with angiotensin-converting enzyme inhibitor (ACEI) or angiotensin receptor blockers (ARBs). On the basis of these previous clinical researches and meta-analyses, the expert consensus document on hypertension from American suggested that ACEI or ARBs should generally be used in hypertensive patients with LVH.^[10]^

However, β-blockers used in majority of the clinical researches were not fat-soluble nor β1-selective. And one newest study conducted by Caglar showed that nebivolol, one of the fat-soluble and selective β1-receptor blockers (FS-β-B), had better effect on regression of LVH than ACEI.^[11]^ We hypothesized that FS-β-B, which including metoprolol, bisoprolol, and nebivolol, reduce left ventricular mass (LVM) to a greater extent than other antihypertensive agents.

The aim of the current network meta-analysis was to compare the efficacy of FS-β-B with other 4 different classes of antihypertensive drugs on LVH regression.

## 2 Methods

### 2.1 Ethical review

All analyses were based on previous published studies, thus no ethical approval and patient consent are required.

### 2.2 Search strategy

We searched PubMed, Web of Science, Cochrane Database, and OVID EBM Reviews (until December 2016) to identify clinical trials only published in English. The search terms included: “left ventricular mass,” “left ventricular hypertrophy,” “regression,” and each class of antihypertensive drugs. For the FS-β-B we also performed searches for each drug separately, such as bisoprolol, nebivolol, and metoprolol. We also manually searched the previously published meta-analyses and bibliographies of the selected publications. Additionally, gray literature was identified by searching the related agencies and clinical trial registers. The reference lists of the original articles and reviews on the topic were examined to identify other eligible studies. A total of 41 randomized controlled trials (RCTs) were included (References supplemental appendix 1–41).

### 2.3 Eligibility criteria

Selection criteria for inclusion in the meta-analysis were as follows: comparison of the effect of antihypertensive drugs, belonging to different drug classes (diuretics, β-blockers, calcium channel blockers [CCBs], ACEI, and ARBs), on left ventricular mass index (LVMI); initiation of drug treatment with monotherapy, with or without add-on therapy for better BP control; no other interventions or treatment, with interruption of all BP-lowering drugs before the run-in period; and availability of echocardiographic LVMI in ≧70% of patients in ≧1 visit after randomization (in case of multiple examinations, the last visit with <70% of analyzable data was taken).

Exclusion criteria were as follows: other β-blockers that were not FS-β-B, such as timolol, propranolol, atenolol, tertatolol, and carvedilol; only reported data of LVM instead of LVMI; hypertensive patients with cardiovascular or renal disease or other clinical conditions, such as diabetes; drug treatments provided for patients were different in all of the treatment arms; treatment duration of <2 months; missing the date of LVM at baseline and during treatment or at baseline with changes from baseline; and age <18 years.

And full publication in a peer-reviewed journal up to December 2016, with the exclusion of data repeats. Two reviewers (XFW and CJL) independently screened the studies to determine whether they satisfied the eligibility criteria. Disagreement between reviewers were resolved by consensus, and a 3rd reviewer (CYL) was consulted when necessary.

### 2.4 Data extraction

Two independent reviewers screened the data from the included studies using a predefined checklist for each study. Disagreements between reviewers were resolved by discussion until a consensus was reached. Data extraction and presentation for this article followed the recommendations of the PRISMA group (References supplemental appendix 1–41). The following data were extracted from each selected study whenever available: demographics and sample characteristics, LVMI, type, treatment duration and dose of antihypertensive drugs, and additional drug used. The primary endpoints in our meta-analysis were regression of LVH, determined by the LVMI.

### 2.5 Data analysis (traditional meta-analysis)

Traditional meta-analysis using the random-effects model was conducted. We computed the pooled mean difference (MD) and 95% credibility interval (CI) as well as the heterogeneity of the included studies. A random-effect model was used to calculate the pooled MD and 95% CI. *I*^2^ statistic was used to indicate the proportion of heterogeneity between studies in total variation; the cut-off points for low, moderate, and high degrees of heterogeneity were 25%, 50%, and 75%, respectively. *I*^2^ value ≤25% indicate no evidence of heterogeneity. Heterogeneity was considered significant when the *P*-value was less than 0.1.^[12]^ If between-study heterogeneity was observed in traditional meta-analysis, then we performed sensitivity analyses by excluding each study individually to explore the possible sources of heterogeneity. The regression analysis based on different duration of medication, treatment regimen (monotherapy or not, double dosage or not), published time, sample size, and study countries were conducted to investigate whether these conditions could influence the results. Traditional meta-analysis was performed with the REVMAN software (version 5.2; Cochrane Collaboration, Oxford, UK) and Stata 12 (StataCorp, College Station, TX).

### 2.6 Data analysis (network meta-analysis)

Network meta-analysis was conducted for mixed treatment comparisons in a Bayesian framework, and the pooled estimates were obtained using the Markov Chains Monte Carlo method. This approach is recommended by the National Institute for Health and Care Excellence (NICE) Decision Support Unit according to the technical support documents on evidence synthesis.^[13,14]^ We performed a random-effects network meta-analysis in GeMTC-GUI-0.14.3, which uses Bayesian Markov Chains Monte Carlo methods^[15,16]^ with 50,000 times random sampling. There were 3 parts in this analysis. First, in the network meta-analysis for the consistency model, we estimated all of the relative effects simultaneously by using the consistency constraint. For example, the parameter dBC was estimated from both direct evidence on BC and indirect evidence on AC and AB. The relative effect results for the consistency model were reported as an MD with a corresponding 95% CI. Then, we estimated the ranking probability for each drug. Rankings regarding treatment efficacy of the 5 drug classes were originally derived from Monte Carlo simulations and presented as the probability of possessing a specific ranking, in which the probabilities of different rankings of the same treatment were summed to 100%.^[17]^ Second, we performed the inconsistency analysis using the inconsistency model and the node-splitting model to check whether the analysis of the trials in the network was indeed consistent. In brief, the inconsistency factors, representing the discrepancy between the direct and indirect evidence, were added to the closed loops of the inconsistency model, that is, dBC = dAC – dABþ + ϕ (ϕ = inconsistency factor). Therefore, the degree of inconsistency, by checking the size of an inconsistency factor within the cycle, was determined for a cycle (eg, ABC) rather than for individual pairwise comparisons.^[18]^ When the 95% CI of the median of the inconsistency factors included zero and if the inconsistency standard deviation was less than or equal to the random-effects standard deviation, the inconsistency can be considered as insignificant. Last, sensitivity analyses were performed to see if the efficacy hierarchies have changed.

## 3 Results

### 3.1 Search results

The search strategy revealed 547 potentially eligible references, and 20 additional records were identified by other means. After the duplicates were removed, 494 studies remained. When the titles were reviewed, 237 studies were excluded. When the abstracts or all content were reviewed in terms of the inclusion and exclusion criteria, 107 studies were excluded. The remaining 41 studies were all included in this meta-analysis (supplemental figure appendix 2). Among these studies, all of them were from journal articles (full manuscripts acquired).

### 3.2 Study characteristics and baseline patient characteristics

**Figure 1:**
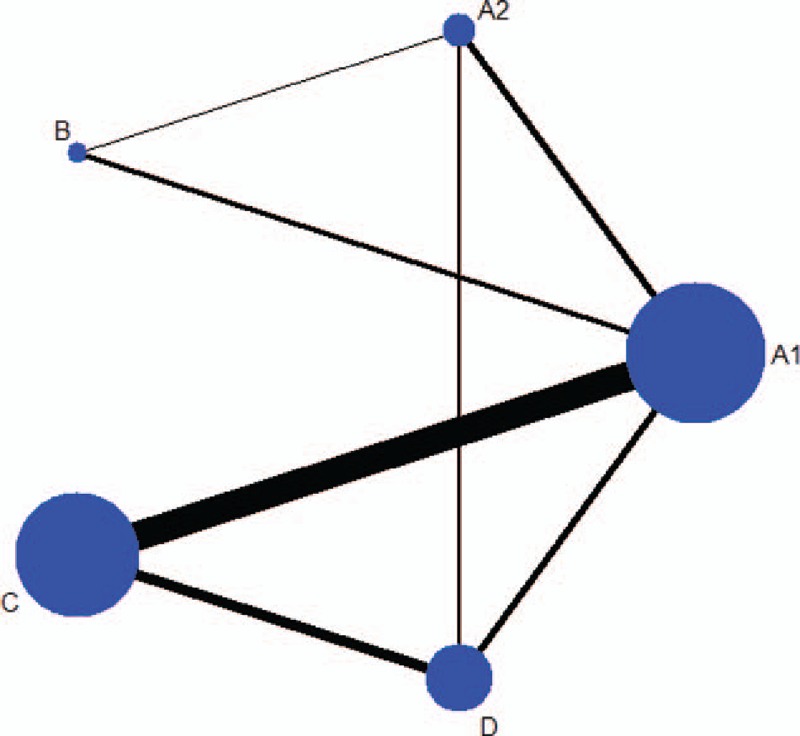
The construction of the network (A1 = ACEI; A2 = ARB; B = FS-β-B; C = CCB; D = diuretic). ACEI = angiotensin-converting enzyme inhibitor, ARB = angiotensin receptor blocker, CCB = calcium channel blocker, FS-β-B = fat-soluble and selective β1-receptor blockers.

Supplemental table appendix 1–2 describes key characteristics of the included studies (design, treatments, follow-up length, and the inclusion criteria of each trial) and the clinical and baseline characteristics of patients enrolled in each trial (age, male, BMI, LVM, etc.). Therapeutic methods in every study were different from each other. There are 22 studies used monotherapy, 13 studies combined with other antihypertension drug, and 6 studies did not mention this. Duration of hypotensor administration was different in these studies, ranging from 2 to 24 months. In these RCTs, 949 patients (36.98%) were assigned to ACEI (perindopril, enalapril, lisinopril, ramipril, etc.); 119 (4.64%) to FS-β-B therapy (atenolol, metoprolol, and nebivolol); 389 (15.16%) to diuretics therapy (hydrochlorothiazide, indapamide, perindopril, etc.); 375 (14.61%) to ARB (eprosartan, telmisartan, etc.); and 708 (27.59%) were randomized to CCB (nimodipine, nitrendipine, etc.). The construction of the network comparisons between different treatment strategies is shown in Fig. 1.

### 3.3 Traditional meta-analyses

**Figure 2:**
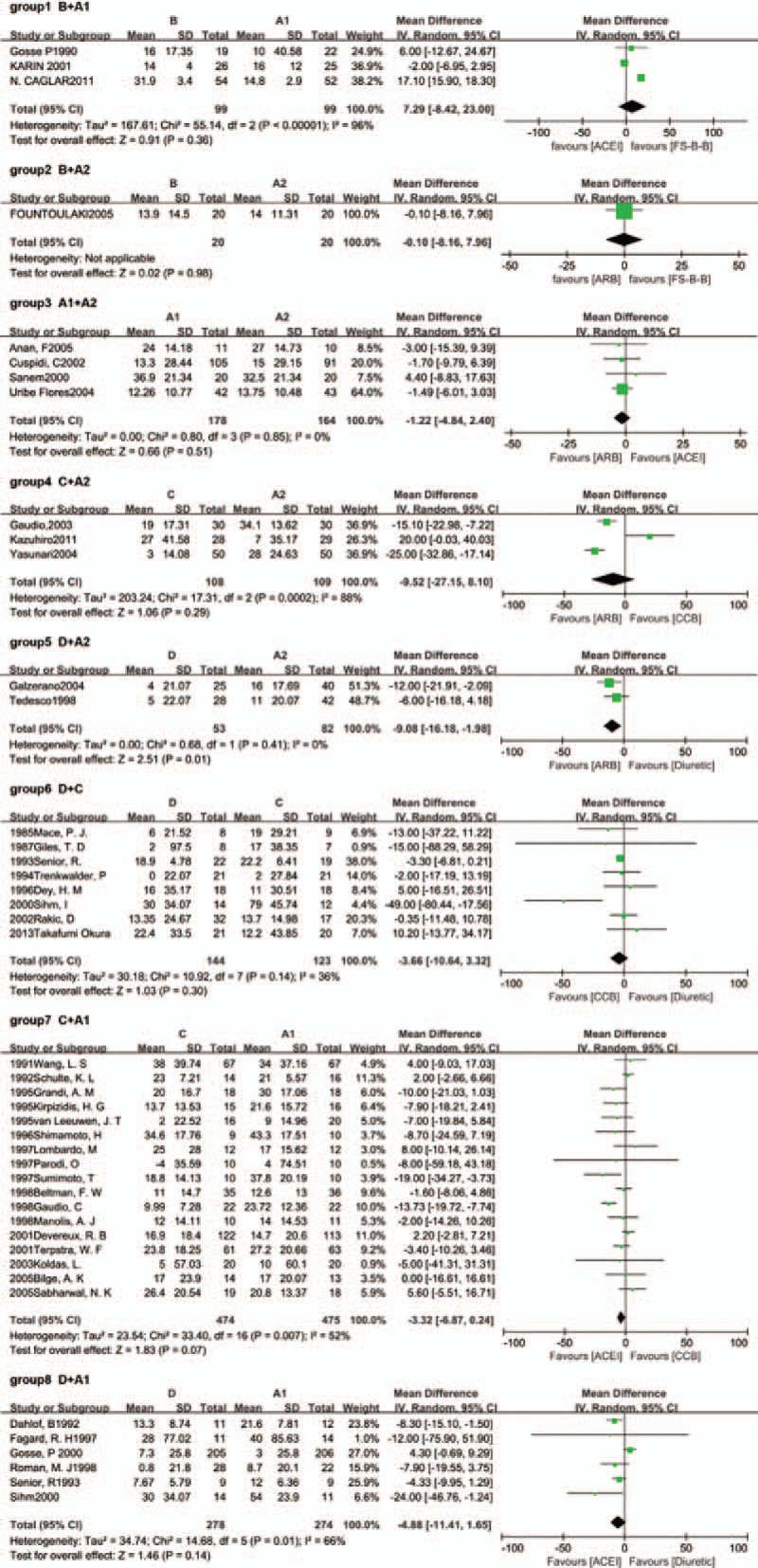
Results of traditional meta-analysis (A1 = ACEI; A2 = ARB; B = FS-β-B; C = CCB; D = diuretic). ACEI = angiotensin-converting enzyme inhibitor, ARB = angiotensin receptor blocker, CCB = calcium channel blocker, FS-β-B = fat-soluble and selective β1-receptor blockers.

Figure 2 (group 1–8) presents the results of the meta-analysis of the data about the regression of LVH between different classes of antihypertension drugs from the 41 included studies. There was not statistical difference between FS-β-B and ACEI (group1; *P* = 0.36). By the way, only 1 study was included in the group 2 (FS-β-B and ARB).

Overall, heterogeneity was moderate, although for several groups the 95% CI included values that showed very high or significant heterogeneity, reflecting the small number of included studies for these pairwise comparison. For example, there were 2 groups which *I*^2^ values were higher than 75%. They were group 1 (FS-β-B vs ACEI, *I*^2^ = 96%) and group 4 (CCB vs ARB, *I*^2^ = 88%). And only 3 studies were included in these groups, respectively.

### 3.4 Bayesian network meta-analyses

**Figure 4:**
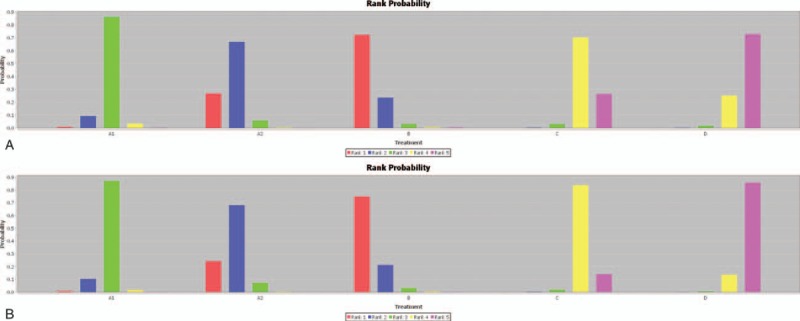
(A) Rangking (A1 = ACEI; A2 = ARB; B = FS-β-B; C = CCB; D = diuretic). (B) Sensitivity analysis of network meta-analysis (A1 = ACEI; A2 = ARB; B = FS-β-B; C = CCB; D = diuretic). ACEI = angiotensin-converting enzyme inhibitor, ARB = angiotensin receptor blocker, CCB = calcium channel blocker, FS-β-B = fat-soluble and selective β1-receptor blockers.

We summarize the results of our random-effects network meta-analysis for the regression of LVH in Table 1. Pooled analysis of all of the included studies indicated that there were no statistical differences between these groups: FS-β-B and ACEI (MD, −7.09; 95% CI, −14.99, 1.27); FS-β-B and ARB (MD, −2.66; 95% Cl, −12.02, 6.31). Although FS-β-B showed greater efficacy when compared with diuretic (MD, 13.04; 95% CI, 3.38, 22.59) or CCB (MD, 10.90; 95% CI, 1.98, 19.49). Figure 4A showed the distribution of probabilities of each treatment being ranked at each of the possible 5 positions. The probabilities of being among the most efficacious treatments were: FS-β-B (72%), ARB (27%), ACEI (0.01%), CCB (0.00%), and diuretic (0.00%) (Table 2).

**Table 1:**
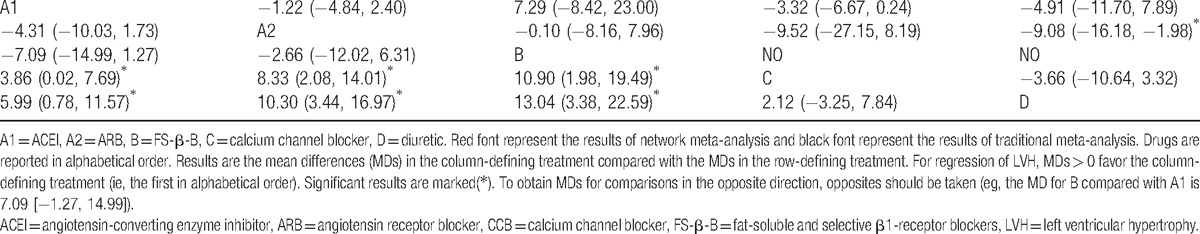
The results of network and traditional meta-analysis for regression of LVH.

**Table 2:**
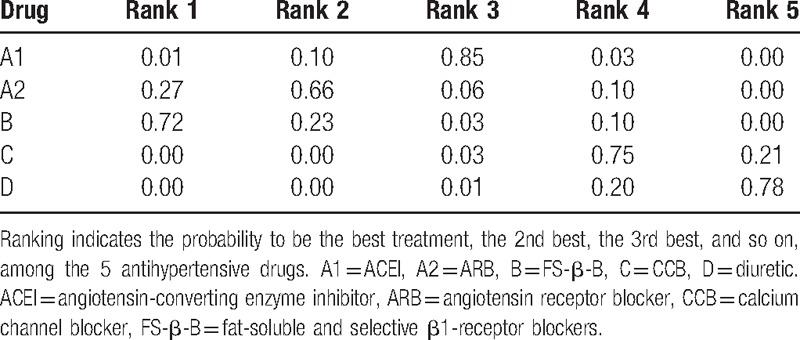
Ranking.

No significant changes of efficacy hierarchies emerged in sensitivity analysis when excluding studies published before 2000, with small sample size(n < 100), carried out in non-western countries, whose follow-up period were less than 1 year, or the study conducted by Gosse (supplemental table appendix 3 and Fig. 4B) (References supplemental appendix 29).

### 3.5 Comparisons between traditional meta-analyses and Bayesian network meta-analyses

Table 1 also presents the results of traditional pairwise meta-analyses. In general, the confidence intervals from traditional pairwise meta-analyses and the CIs from Bayesian network meta-analyses overlapped. By comparing with the results obtained from the Bayesian network meta-analysis, the results of the traditional meta-analysis were largely comparable.

### 3.6 Heterogeneity (traditional meta-analyses)

**Figure 3:**
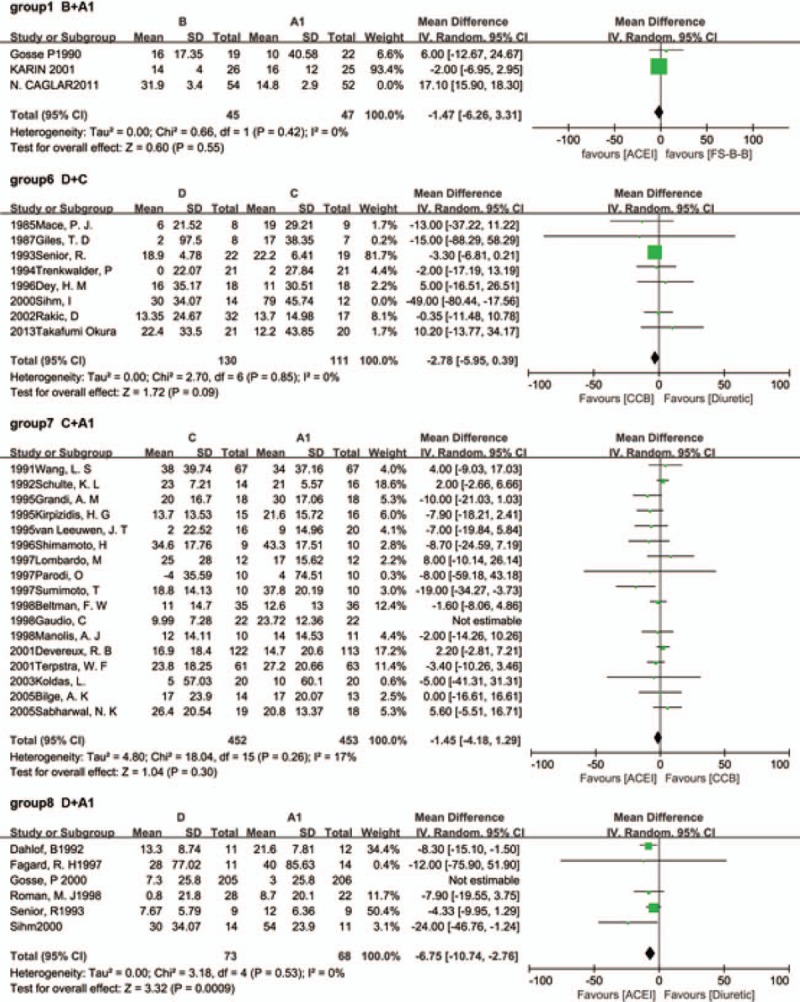
Sensitive analysis of traditional meta-analysis (A1 = ACEI; A2 = ARB; B = FS-β-B; C = CCB; D = diuretic). ACEI = angiotensin-converting enzyme inhibitor, ARB = angiotensin receptor blocker, CCB = calcium channel blocker, FS-β-B = fat-soluble and selective β1-receptor blockers.

We performed sensitivity analyses by excluding each study individually to explore the possible sources of heterogeneity (Fig. 3). When we repeated the analysis after excluding the study conducted by Sihm in the group6 (diuretic vs CCB) (References supplemental appendix 36), the study conducted by Gaudio in the group 7 (CCB vs ACEI) (References supplemental appendix 16) and the study conducted by Caglar in the group 1 (FS-β-B vs ACEI) (References supplemental appendix 1), we found that the pooled effect did not change, and the between-study heterogeneity decreased significantly (*I*^2^ = 36.0% to 0%, *I*^2^ = 52.0% to 17%, and *I*^2^ = 96.0% to 0%, respectively). These 3 studies could be the source of the heterogeneity. First, in the group 2, the study conducted by Sihm was the only one that added other antihypertensive drugs into original drug therapy. Second, the study conducted by Gaudio was the only one that employed magnetic resonance imaging instead of echocardiography to measure the LVMI in the group 3. Last, the cause of the significant heterogeneity in the group 1 might lie in the limitation of the study number included. Just only 3 studies were included.

Besides, when we excluded the study conducted by Gosse in the group 8 (References supplemental appendix 29), the pooled result changed and the between-study heterogeneity decreased significantly (*P* = 0.16–0.001; *I*^2^ = 73%–5%). The possible causes were listed as follows. First, the drug used in this study was indapamide rather than hydrochlorothiazide. They were grouped together in a single class of antihypertensive drug (diuretic). Indapamide had calcium-antagonistic effect, not only diuretic effect. Second, a total of 131 patients (25.9%) prematurely discontinued the study. The missing rate was higher than other studies in group 8. Considering the significant heterogeneity, we performed sensitivity analysis by excluding this study in our network meta-analysis.

Moreover, the regression analysis based on different duration of medication, treatment regimen, published time, sample size, and study countries showed that there was no one factor influenced our results (supplemental table appendix 4).

### 3.7 Model inconsistency (Bayesian network meta-analyses)

In the network meta-analysis, the disagreement between direct and indirect comparison was concerning and was examined by calculating the inconsistency factors. For all comparisons in the regression of LVH, the 95% CI of inconsistency factors from all cycles included zero (Table 3), and the node-splitting method showed no significant inconsistency within the networks for any of these outcomes, which suggested that the results in the network were consistent between direct and indirect evidence (Table 4).

**Table 3:**
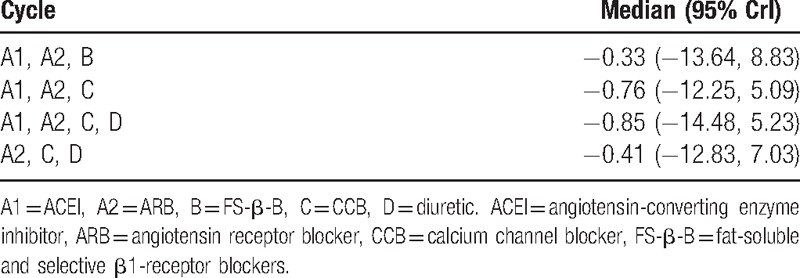
Inconsistency factors.

**Table 4:**
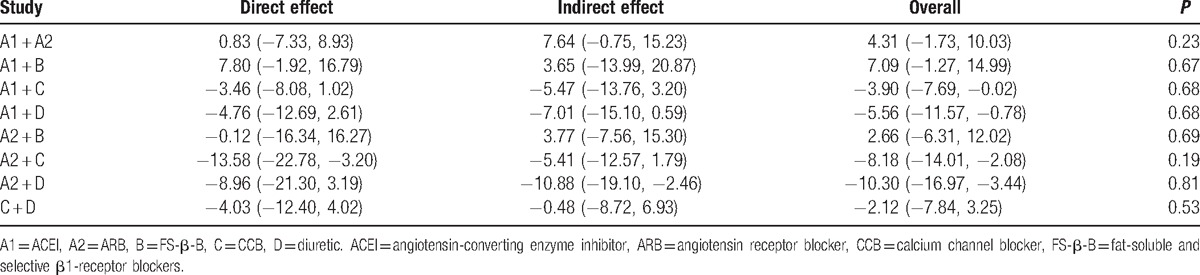
The results of node-splitting method.

## 4 Discussion

To our knowledge, this is the first and only one Bayesian network meta-analysis that included most updated studies to evaluate all of the 5 classes of antihypertensive drugs on regression of LVH. The key point of this analysis was whether the accepted idea, β-blockers were associated with less regression for LVH patients than ACEI or ARB, was right. Using the network meta-analysis of randomized controlled trials, the indirect comparisons between drugs were made possible, and the relative differences between different classes of antihypertensive agents could be determined. In this Bayesian network meta-analysis, the probability ranking analysis suggested that FS-β-B was the preferred agent for the regression of LVH.

The mechanisms underlying the beneficial effects of FS-β-B remain unknown and may be multifactorial. First, adrenergic system plays an important role in the development of LVH and heart failure (HF).^[19,20]^ Simply, adrenergic receptors belong to the guanine nucleotide-binding G protein-coupled receptor (GPCR) superfamily. So far, 7 mammalian isoforms of GPCR kinases (GRK1–GRK7) have been identified. GRK2 and GRK5 are the predominantly expressed isoforms in the heart. Both of them could inhibit NF-kB transcriptional activity which was relevant in the development of LVH.^[21]^ Second, according to a recently published updated clinical and pharmacological evaluation edited by Maung-U,^[22]^ there were several differences between FS-β-B and other β-blockers. On the one hand, lipophilic compounds are rapidly adsorbed in the gastrointestinal tract and cell membrane, and are extensively metabolized in the liver (1st-pass metabolism), resulting in a shorter half life, a faster response time when compared to other β-blockers.^[23]^ On the other hand, β1-receptors mainly exist in heart, while β2-receptors mainly exist in bronchus and vascular smooth muscle. The reduced inhibitory effect on β2-receptor makes the selective β1-blockers less likely to cause peripheral vasoconstriction, so that it could bring better antihypertensive effect than other β-blockers. Third, previous fundamental research showed that cardiac-specific overexpression of β1-receptors in mice caused cardiomyocyte hypertrophy.^[24,25]^ However, the consequences of overexpression of β2-receptors were more complex. A 200-fold overexpression of β2-receptors in the murine heart was accompanied by increased heart rate and left ventricular contractility.^[26]^ A 350-fold overexpression of β2-receptors in mouse models was associated with dilated cardiomyopathy, heart failure, and mortality.^[27]^ For these reasons, selective β1-receptor blockers might show better regression on LVH. Last, the more pronounced effect of FS-β-B may not be ascribed only to the reduction of blood pressure, but other factors might have concurred. For example, nebivolol, a new generation β-receptor blocker, had a vasodilator property that mediated by the L-arginine/NO pathway. Besides, differently from classical β-blockers, nebivolol has been demonstrated to have antiproliferative activity,^[28–32]^ attributable to the increase of NO bioavailability also at coronary and cardiac level.^[33,34]^ NO is involved on LV fibrotic component regression.^[32,35]^ This property might have played an important role in the regression of LV fibrotic component, that characterizes LVH.^[36,37]^ In addition, nebivolol reduces large arterial stiffness and central blood pressure,^[38,39]^ which have a pathogenetic role in promoting LVH.^[39,40]^ One previous meta-analysis concluded that nebivolol achieved similar or better rates of treatment response and BP normalization than other drug classes, with significantly better tolerability than losartan, other β-blockers, and all antihypertensive drugs combined. This meta-analysis suggested that nebivolol, one of the FS-β-B, is likely to have advantages over existing antihypertensives and may have a role in the 1st-line treatment of hypertension.^[41]^

The information revealed in our meta-analysis will be useful for clinicians and will enable them to select the optimal antihypertensive agents to regress the LVH in hypertensive patients. Especially in Asia, where LVH caused by hypertension was common.^[42,43]^

## 5 Conclusion

In our study, FS-β-B were estimated to have a 72% chance of being the best for regression of LVH. Although there were no statistical difference between FS-β-B and ARB/ACEI. The clinical evidence related to the FS-β-B in regression of LVH was insufficient considering the limitation of the study number. So, more studies are needed with FS-β-B to find out if they do indeed reduce LVM to a greater extent than other antihypertensive agents do and if this effect would lead to a better prognosis.

## 6 Limitation

As with any meta-analysis, several limitations should be highlighted. First, there were significant heterogeneity in group 4 (CCB vs ARB) in traditional meta-analyses. It might be contributed to the limitation of the study number considering that only 3 studies were included in these groups. So, even after we performed sensitivity analysis, we could not find out the sources of heterogeneity in group 4. Second, most of the patients were prescribed with different treatment regimens, such as the dosage, combination antihypertensive drugs, and duration. Our results were influenced inevitably by these confounding factors. Although we conducted regression analysis to control these factors. Third, different drugs were grouped together in a single class of antihypertensive drug, such as indapamide and hydrochlorothiazide. And that might be the reason why the study conducted by Gosse brought about significant heterogeneity and influenced the result of traditional meta-analysis in group 8. Although no significant change in efficacy hierarchies emerged in sensitivity analysis after excluding the study conducted by Gosse. Last, network meta-analysis was simply a statistical method, and its clinical literature evidence level might not be that good. But, the point was the clinical significance it reflected, especially for the question that nobody paid attention to.

## Acknowledgments

The authors thank the assistant of the statistical analysis from the Yilong Education, Inc.

## Supplementary Material

Supplemental Digital Content

## Supplementary Material

Supplemental Digital Content
