# Real role of β-blockers in regression of left ventricular mass in hypertension patients: Bayesian network meta-analysis: Erratum

**DOI:** 10.1097/MD.0000000000010991

**Published:** 2018-05-25

In the article, “Real role of β-blockers in regression of left ventricular mass in hypertension patients: Bayesian network meta-analysis”,^[1]^ which appeared in Volume 96, Issue 10 of *Medicine*, Drs FuWei Xing, Jingzhou Jiang, Anli Tang and Yili Chen should also be affiliated with Key Laboratory on Assisted Circulation, Ministry of Health, Guangzhou 510080, China.
