# Supplementary material for: Real role of β-blockers in regression of left ventricular mass in hypertension patients: Bayesian network meta-analysis
Source: Medicine (Baltimore). 2017 Mar 10;96(10):e6290. doi: 10.1097/MD.0000000000006290 (PMC5348202; doi:10.1097/MD.0000000000006290)

Fig appendix 1 Selection process [Ra = References(appendix); A1 = ACEI; A2 = ARB; B = fat-soluble and selective β1-receptor; C = CCB; D = Diuretic]


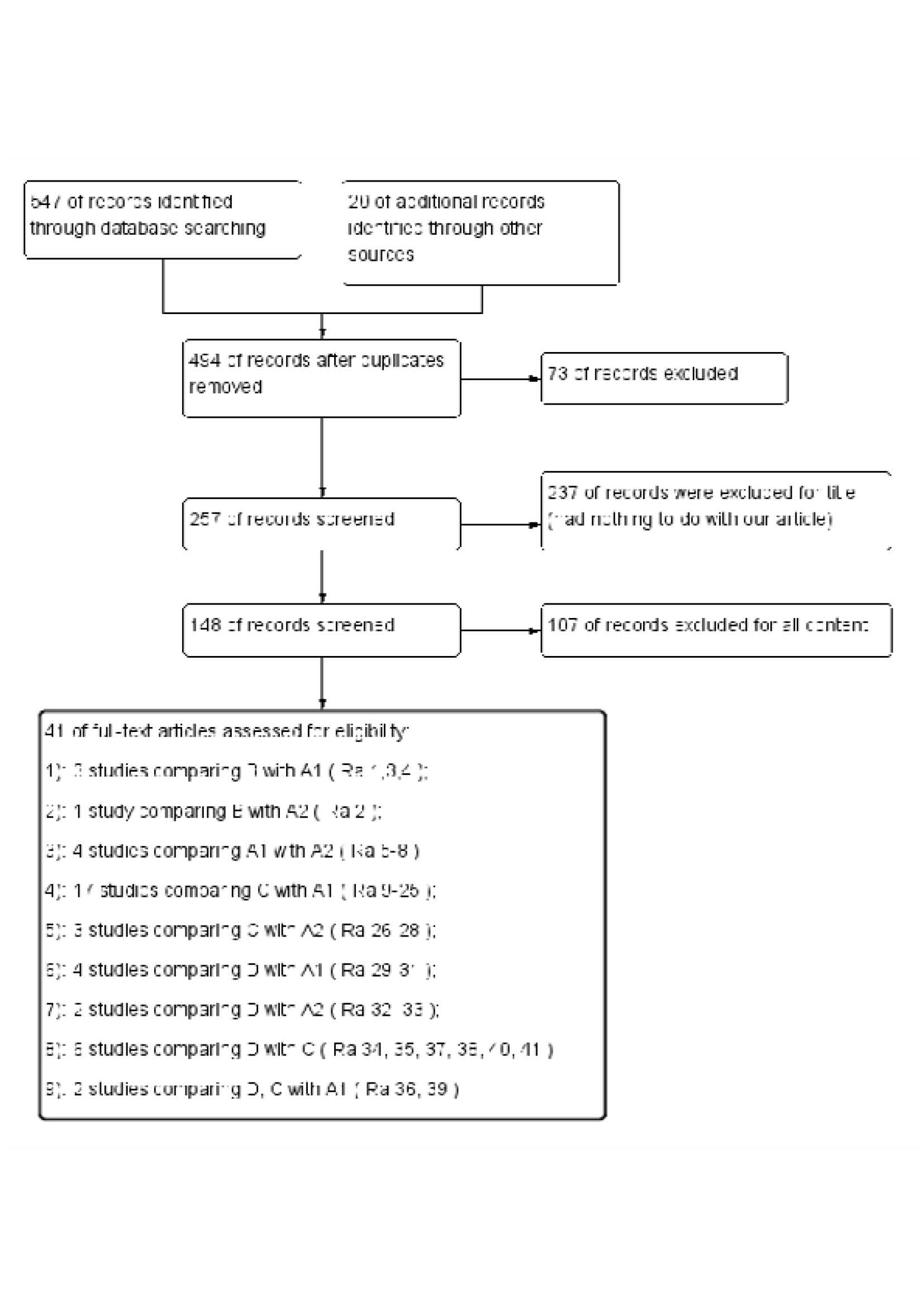

Supplement: Supplemental Digital Content [file medi-96-e6290-s001.doc]
