# Supplementary material for: Real role of β-blockers in regression of left ventricular mass in hypertension patients: Bayesian network meta-analysis
Source: Medicine (Baltimore). 2017 Mar 10;96(10):e6290. doi: 10.1097/MD.0000000000006290 (PMC5348202; doi:10.1097/MD.0000000000006290)
Supplement: Supplemental Digital Content [file medi-96-e6290-s002.doc]

Table appendix1( C + A1 )：The characteristics of the included studies

| Study（C+A1） | Design | Center | Drug(C) | Dose(mg) | Drug(A1) | Dose(mg) | Inclusion criteria | Time | Other drug |
| --- | --- | --- | --- | --- | --- | --- | --- | --- | --- |
| Bilge,2005 | NR - RCT | Single | Amlodipine | 5 - 10 | Fosinopril | 10 - 20 | HT(untreated,mild - moderate) | 24W | Monotherapy |
| Sabharwal,2005 | DB - RCT | Single | Nifedipine | 20 - 40 * bid | Imidapril | 5 - 10 | HT(untreated)+LVH | 24W | HCTZ |
| Koldas,2003 | NR - RCT | Single | Nifedipine | 20 | Perindopril | 4 | HT(mild - moderate)+LVH | 12W | Monotherapy |
| Devereux,2001 | DB - RCT | Mult | Nifedipine | 30 - 60 | Enalapril | 10 - 20 | HT+LVH | 48W | HCTZ/Atenolol |
| Terpstra,2001 | DB - RCT | Mult | Amlodipine | 5 - 10 | Lisinopril | 10 - 20 | HT(untreated,mild - moderate) | 96W | NR |
| Beltman,1998 | DB - RCT | Single | Amlodipine | 5 - 10 | Lisinopril | 10 - 20 | HT(untreated,25 - 75 years) | 48W | Monotherapy |
| Gaudio,1998 | PROBE | Single | Nitrendipine | 20 - 40 | Benazepril | 10 - 20 | LVH+HT(untreated,poor control/tolerance) | 24W | Monotherapy |
| Manolis,1998 | DB - RCT | Single | Isradipine | 5 | Spirapril | 6 | HT+LVH | 24W | Monotherapy |
| Lombardo,1997 | PROBE | Single | Amlodipine | 5 - 10 | Fosinopril | 10 - 20 | HT(mild - moderate) | 48W | Doxazosin |
| Parodi,1997 | DB - RCT | Single | Verapamil | 240 - 480 | Enalapril | 10 - 40 | HT+normal coronary arteries | 24W | Monotherapy |
| Sumimoto,1997 | NR - RCT | Single | Nicardipine | 40 - 80 * bid | Alacepril | 25 - 100 * bid | HT +LVH | 72W | NR |
| Shimamoto,1996 | SB - RCT | Single | Nifedipine | 10 - 30 | lisinopril | 10 - 30 | HT(stage II)+LVH | 48W | Monotherapy |
| Grandi,1995 | NR - RCT | Single | Isradipine | 5 | Perindopril | 4 - 8 | HT +LVH+normal LV diastolic diameter | 24W | NR |
| Kirpizidis,1995 | DB - RCT | Single | Nifedipine | 20 * bid | Fosinopril | 20 | HT +LVH | 24W | HCTZ |
| van Leeuwen,1995 | DB - RCT | Mult | Diltiazem | 300 | lisinopril | 10 - 20 | HT(mild - moderate,untreated, 25 - 70 years) | 24W | Monotherapy |
| Schulte,1992 | DB - RCT | Single | Nifedipine | 40 - 80 | Perindopril | 4 - 8 | HT(mild - moderate,untreated/insufliciently treated) | 24W | HCTZ |
| Wang, 1991 | NR - RCT | Single | Nitrendipine | 20 - 40 | Captopril | 75 - 150 | HT +LVH | 96W | Monotherapy |

NR = not reference; RCT = randomized controlled trial; SB = single blinded; DB = double blinded; Mult = Multi-center; HT = hypertension; LVH = left ventricular hypertrophy; HCTZ = hydrochlorothiazide; W = weeks；PROBE = prospective randomized open design with blinded endpoint evaluation.

A1 = ACEI; A2 = ARB; B = fat-soluble and selective β1-receptor blockers; C = Calcium channel blocker; D = Diuretic.

Table appendix1 ( A1 + A2 )： The characteristics of the included studies

| Study（A1+A2） | Design | Center | Drug(A1) | Dose(mg) | Drug(A2) | Dose(mg) | Inclusion criteria | Time | Other drug |
| --- | --- | --- | --- | --- | --- | --- | --- | --- | --- |
| Anan,2005 | NR | Single | perindopril | 4 - 8 | Valsartan | 80 - 160 | HT(untreated) | 32W | Indapamide |
| Uribe 2004 | NR | Single | Enalapril | 20 | Losartan | 100 | HT(moderate)+LVH | 24W | NR |
| Akinboboye,2000 | NR | Single | lisinopril | 10 | losartan | 50 | HT+LVH | 48W | Diuretics/Atenolol |
| Nalbantgil,2000 | DB - RCT | Single | Enalapril | 20 | Valsartan | 80 | HT(mild - moderate) | 24W | Monotherapy |

Table appendix1( B + A1 and B + A2 )： The characteristics of the included studies

| Study（B+A1） | Design | Center | Drug（B） | Dose(mg) | Drug(A1) | Dose(mg) | Inclusion criteria | Time | Other drug |
| --- | --- | --- | --- | --- | --- | --- | --- | --- | --- |
| Caglar,2011 | PROBE | Mult | Nebivolol | 5 | Ramipril | 2.5 | HT+LVH | 39W | Indapamide/HCTZ |
| Malmqvist,2001 | PROBE | Single | Metoprolol | 50 | Captopril | 50 | HT(untreated) | 48W | HCTZ/Furosemid/CCB |
| Gosse,1990 | SB - RCT | Single | Bisoprolol | 10 | Enalapril | 20 | HT(sustained ) | 24W | Monotherapy |
| Study（B+A2） |  |  | Drug（B） |  | Drug(A2) |  |  |  |  |
| Fountoulaki,2005 | SB - RCT | Single | Nebivolol | 2.5 - 5.0 | Telmisartan | 40 - 80 | HT(mild - moderate) | 12W | Indapamide |

Table appendix1 ( D + A1 )： The characteristics of the included studies

| Study（D+A1） | Design | Center | Drug(D) | Dose(mg) | Drug(A1) | Dose(mg) | Inclusion criteria | Time | other drug |
| --- | --- | --- | --- | --- | --- | --- | --- | --- | --- |
| Gosse,2000 | DB - RCT | Mult | Indapamide | 1.5 | Enalapril | 20 | HT+LVH(>20 years) | 48W | Monotherapy |
| Sihm,2000 | SB - RCT | Mult | Amiloride or  HCTZ | 2.5  25 | Perindopril | 4 - 8 | HT(poor control and was discontinued 6W) | 48W | Atenolol/Isradipine/Hydralazine |
| Roman,1998 | DB - RCT | Single | HCTZ | 12.5 - 50 | Ramipril | 5 - 20 | HT(untreated/following a placebo washout period) | 24W | Monotherapy |
| Senior,1993 | DB - RCT | Single | Indapamine | 2.5 | Enalapril | 20 | HT+LVH(20 - 75 years) | 24W | Monotherapy |
| Dahlof,1992 | DB - RCT | NR | HCTZ | 12.5 - 50 | Enalapril | 10 - 40 | HT(untreated,males,20 - 65 years) | 56 - 72W | Monotherapy |

Table appendix1( D + A2 and C + A2 ) ： The characteristics of the included studies

| Study（D+A2） | Design | Center | Drug(D) | Dose(mg) | Drug(A2) | Dose(mg) | Inclusion criteria | Time | Other drug |
| --- | --- | --- | --- | --- | --- | --- | --- | --- | --- |
| Galzerano,2004 | DB - RCT | Single | HCTZ | 25 | Telmisartan | 80 | HT(mild - moderate,≥18 years) | 48W | Monotherapy |
| Tedesco,1998 | DB - RCT | Single | HCTZ | 25 | losartan | 50 | HT(mild - moderate) | 88W | NR |
| Study（C+A2） |  |  | Drug(C) |  | Drug(A2) |  |  |  |  |
| Yamamoto,2011 | PROBE | Single | Amlodipine | 2.5 - 5.0 | Losartan | 50 - 100 | HT(mild - moderate)+LVH+diastolic dysfunction and preserved systolic function. | 72W | Other medication,with the exception of ARB,CCB,ACEI or β - B |
| Yasunari,2004 | DB - RCT | Single | Amlodipine | 5 | Valsartan | 80 | HT(untreated,discontinued drugs) | 32W | Monotherapy |
| Gaudio,2003 | PROBE | Double | Amlodipine | 5 - 10 | Irbesartan | 150 - 300 | HT(untreated,≦80years)+LVH | 24W | Monotherapy |

Table appendix1( D + C )： The characteristics of the included studies

| Study（D+C） | Design | Center | Drug(D) | Dose(mg) | Drug(C) | Dose(mg) | Inclusion criteria | Time | Other drug |
| --- | --- | --- | --- | --- | --- | --- | --- | --- | --- |
| Okura,2013 | NR | Mult | HCTZ | 12.5 | Amlodipine or other | NR | LVH+HT(poor treated by ARB) | 48W | losartan，and other(non - ARB,CCB,and diuretic) |
| Rakic,2002 | DB - RCT | Single | Indapamide or  Chlorthalidone | 2.5  25 | Nicardipine | 20 * 3 | LVH+HT(poor treated or newly discovered,20 - 75 years) | 24W | NR |
| Sihm,2000 | SB - RCT | Mult | Amiloride or  HCTZ | 2.5  25 | Isradipine | 2.5 - 10 | HT(poor control and was discontinued 6W) | 48W | Atenolol,Isradipine,Hydralazine |
| Dey,1996 | DB - RCT | Mult | HCTZ | 12.5 - 50 | Nifedipine | 30 - 120 | HT(StageI - III,diastolic) | 13W | Monotherapy |
| Senior,1993 | DB - RCT | Single | indapamine | 2.5 | Nifedipine | 40 | HT+LVH(20 - 75 years) | 24W | Monotherapy |
| Trenkwalder,1994 | DB - RCT | Single | HCTZ or  Triamterene | 25  50 | felodipine | 5 | HT(≥70 years,untreated) and without CAD | 12W | Monotherapy |
| Giles,1987 | DB - RCT | Single | HCTZ | 50 | nitredipine | 20 | HT(≥50 years,mild or moderate) | 8W | Monotherapy |
| Mace,1985 | NR | Single | Indapamide | 2.5 | Nifedipine | 10 | HT | 16W | Monotherapy |

Table appendix2 ( C + A1 )：The clinical and baseline characteristics of patients

| Study | Sample | Men | Age | | BMI | | LVMI | | SBP | | DBP | | HR | | PWT | |
| --- | --- | --- | --- | --- | --- | --- | --- | --- | --- | --- | --- | --- | --- | --- | --- | --- |
| C + A1 | C A1 | C A1 | C | A1 | C | A1 | C | A1 | C | A1 | C | A1 | C | A1 | C | A1 |
| Bilge, 2005 | 14 + 13 | 8 + 9 | 46 ± 6 | 49 ± 10 | 25.9 ± 3.7 | 26.8 ± 3.7 | 122 ± 26 | 118 ± 23 | 151 ± 15 | 161 ± 16 | 101 ± 6 | 103 ± 7 | 71 ± 6 | 74 ± 8 | NR | NR |
| Sabharwal,2006 | 19 + 18 | 31 + 29 | 54.7 ± 9.15 | 52.2 ± 10.92 | NR | NR | 148 ± 31 | 146 ± 25 | 153 ± 15 | 149 ± 12 | 107 ± 7 | 106 ± 7 | 79 ± 12 | 70 ± 10 | NR | NR |
| Koldas, 2003 | 20 + 20 | T39 | 59 ± 13 | 60 ± 15 | NR | NR | 203 ± 56 | 202 ± 62 | 175 ± 27 | 180 ± 25 | 95 ± 13 | 95 ± 15 | NR | NR | NR | NR |
| Devereu，2001 | 122 + 113 | 101 + 98 | 63.0 ± 8.6 | 63.5 ± 9.0 | NR | NR | 133.3 ± 25.2 | 130.9 ± 25.1 | 171.0 ± 20.7 | 172.0 ± 21.1 | 98.2 ± 10.2 | 97.7 ± 10.1 | 73.3 ± 11.2 | 73.3 ± 13.1 | 10.3 ± 1.31 | 10.4 ± 1.2 |
| Terpstra, 2001 | 61 + 63 | 38 + 54 | 67 ± 4 | 67 ± 4 | 28.2 ± 3.4 | 28.4 ± 4.2 | 109.1 ± 19.6 | 114.1 ± 23.3 | 175 ± 15 | 17 ± 14# | 92 ± 8 | 93 ± 9 | NR | NR | NR | NR |
| Beltman, 1998 | 35 + 36 | 18 + 26 | 53 ± 10 | 54 ± 11 | 27.2 ± 4.3 | 27.8 ± 3.4 | 87.5 ± 21.1 | 90.6 ± 16.2 | 158 ± 16 | 161 ± 15 | 102 ± 5 | 100 ± 4 | 68.6 9.2 | 68.7 8.2 | NR | NR |
| Gaudio, 1998 | 25 + 25 | 17 + 15 | 52.9 ± 9.8 | 54.2 ± 9.6 | NR | NR | 139.1 ± 6.7 | 146.6 ± 12.4 | 160 ± 12 | 158 ± 13 | 102 ± 4 | 103 ± 4 | 76 ± 8 | 79 ± 4 | 10.9 ± 0.61* | 11.1 ± 0.65* |
| Manolis, 1998 | 10 + 11 | 13 + 14 | NR | NR | NR | NR | 140 ± 15 | 139 ± 15 | 227 ± 14 | 221 ± 16 | NR | NR | NR | NR | NR | NR |
| Lombardo, 1997 | 12 + 12 | 12 + 11 | 44.6 ± 9.8 | 48.2 ± Z9 | 25.5 ± 1.6 | 25.5 ± 2.2 | 125 ± 32 | 106 ± 18 | 141.3 ± 12.8 | 137.2 ± 8.4 | NR | NR | 79.3 ± 10.6 | 77.1 ± 9.0 | 10.1 ± 1.5 | 9.8 ± 1.5 |
| Parodi, 1997 | 10 + 10 | 7 + 6 | 54 ± 6 | 52 ± 8 | NR | NR | 162 ± 37 | 164 ± 75 | 167 ± 11 | 164 ± 16 | 105 ± 11 | 103 ± 6 | 65 ± 8 | 63 ± 10 | NR | NR |
| Sumimoto, 1997 | 10 + 10 | 6 + 5 | 61.3 ± 7.6 | 53.9 ± 7.4 | NR | NR | 133.2 ± 11.7 | 137.1 ± 14.8 | 176.0 ± 13.9 | 168.2 ± 22.3 | 97.0 ± 5.3 | 99.0 ± 5.5 | 73.8 ± 14.6 | 71.6 ± 9.7 | NR | NR |
| Shimamoto, 1997 | 9 + 10 | T16 | 67.7 ± 5.9 | 68.6 ± 6.2 | NR | NR | 174.9 ± 16.5 | 175.2 ± 15.5 | 173.9 ± 12.7 | 175.8 ± 15.4 | 94.9 ± 7.8 | 95.0 ± 9.2 | 69.9 ± 9.2 | 68.9 ± 10.3 | NR | NR |
| Grandi, A.1997 | 18 + 18 | 9 + 9 | 45 ± 5 | 44 ± 7 | 24.7 ± 0.8 | 24.9 ± 0.5 | 139 ± 15 | 142 ± 14 | 155 ± 8 | 156 ± 7 | 106 ± 7 | 105 ± 6 | 68 ± 6 | 71 ± 7 | NR | NR |
| Kirpizidis,1995 | 15 + 16 | 4 + 4 | 61.2 ± 4.2 | 59.1 ± 3.3 | NR | NR | 146.4 ± 14 | 145.5 ± 17 | NR | NR | 103.6 ± 6 | 102.8 ± 7 | 72 ± 9 | 73 ± 8 | 11.6 ± 0.9 | 11.4 ± 0.8 |
| van, 1996 | 16 + 20 | NR | 52.8 ± 8.6 | 47.4 ± 7.9 | 26.9 ± 2.8 | 26.3 ± 2.9 | NR | NR | NR | NR | NR | NR | NR | NR | NR | NR |
| Schulte, 1992 | 14 + 16 | 12 + 17 | NR | NR | NR | NR | 141 ± 6 | 148 ± 5 | 149 ± 4 | 157 ± 4 | 104 ± 2 | 106 ± 2 | 77 ± 2 | 77 ± 3 | 9.9 ± 0.4 | 10.6 ± 0.3 |
| Wang, 1991 | 67 + 67 | 47 + 43 | 48 ± 11 | 47 ± 12 | NR | NR | 148 ± 42 | 144 ± 35 | 164 ± 29 | 168 ± 27 | 97 ± 14 | 94 ± 11 | NR | NR | 14.2 ± 1 | 14 ± 1.1 |

BMI = Body Mass Index; LVMI = left ventricular mass index; SBP = systolic blood pressure; DBP = diastolic blood pressure; HR = heart rate; PWT = left ventricular posterior wall thickness; # means the data was the same with data from original paper, but it might be wrong because of the author’s negligence;  * means we had the data of mean wall thickness instead of the data of PWT ; ** means we had the data of relative wall thickness instead of the data of PWT ;T = total number.

A1 = ACEI; A2 = ARB; B = fat-soluble and selective β1-receptor blockers; C = Calcium channel blocker; D = Diuretic.

Table appendix2( A1 + A2 )： The clinical and baseline characteristics of patients

| Study | Sample | Men | Age | | BMI | | LVMI | | SBP | | DBP | | HR | | PWT | |
| --- | --- | --- | --- | --- | --- | --- | --- | --- | --- | --- | --- | --- | --- | --- | --- | --- |
| A1 + A2 | A1 A2 | A1 A2 | A1 | A2 | A1 | A2 | A1 | A2 | A1 | A2 | A1 | A2 | A1 | A2 | A1 | A2 |
| Anan, 2005 | 11 + 10 | 6 + 4 | 59 ± 7 | 59 ± 8 | 25.4 ± 1 | 25.5 ± 1 | 149 ± 16 | 151 ± 16 | 156 ± 9 | 157 ± 7 | 97 ± 6 | 97 ± 7 | 70 ± 7 | 72 ± 8 | 11.9 ± 0.3 | 11.7 ± 0.5 |
| Uribe, 2004 | 42 + 43 | 11 + 14 | 46.24 ± 5.91 | 48.37 ± 6.06 | 26.13 ± 1.29 | 25.87 ± 1.61 | 127.93 ± 11.03 | 131.56 ± 10.12 | 149.36 ± 7.54 | 151.15 ± 5.61 | 99.38 ± 6.04 | 100.93 ± 5.04 | NR | NR | NR | NR |
| Cuspidi, 2002 | 105 + 91 | 69 + 53 | 52.8 ± 9.4 | 53.1 ± 9.6 | 26.2 ± 2.9 | 26.1 ± 3.3 | 143.4 ± 27.5 | 141.0 ± 24.1 | 162.4 ± 8.9 | 163.1 ± 9.7 | 101 ± 4.4 | 101.5 ± 3.9 | 73.0 ± 10.4 | 75.7 ± 9.6 | 10.0 ± 1.2 | 10.1 ± 0.9 |
| Nalbantgil, 2000 | 20 + 20 | NR | 53.4 ± 5.5 | 54.8 ± 5.2 | NR | NR | 165.0 ± 24.2 | 162.1 ± 22.4 | 164.8 ± 7.7 | 166.4 ± 7.8 | 100.3 ± 3.3 | 101.1 ± 3.6 | NR | NR | 13.2 ± 0.6 | 13.4 ± 0.7 |

Table appendix2( B + A1 and B + A2)： The clinical and baseline characteristics of patients

| Study | Sample | Man | Age | | BMI | | LVMI | | SBP | | DBP | | HR | | PWT | |
| --- | --- | --- | --- | --- | --- | --- | --- | --- | --- | --- | --- | --- | --- | --- | --- | --- |
| B + A1 | B A1 | B A1 | B | A1 | B | A1 | B | A1 | B | A1 | B | A1 | B | A1 | B | A1 |
| Caglar, 2011 | 54 + 52 | 26 + 25 | 50.4 ± 0.89 | 51.1 ± 0.87 | 30.1 ± 0.54 | 28.8 ± 0.58 | 149.6 ± 2.6 | 145.2 ± 2.7 | 161 ± 1.6 | 158 ± 1.5 | 99.8 ± 1.1 | 99.0 ± 0.7 | 75.3 ± 0.6 | 75.8 ± 1.1 | 0.492 ± 0.007** | 0.489 ± 0.012** |
| Malmqvist, 2001 | 26 + 25 | 11 + 18 | 51 ± 8 | 50 ± 7 | 26.4 ± 3.5 | 25.4 ± 2.9 | 116 ± 19 | 113 ± 23 | 158 ± 14 | 159 ± 13 | 101 ± 8 | 103 ± 7 | NR | NR | 11.6 ± 1.5 | 11.6 ± 1.6 |
| Gosse, 1990 | 19 + 22 | 15 + 22 | 51 ± 11 | 49 ± 11 | NR | NR | 141 ± 19 | 143 ± 42 | 172 ± 16 | 170 ± 17 | 106 ± 8 | 104 ± 9 | 81 ± 13 | 73 ± 12 | 10.3 ± 1.4 | 9.9 ± 2.0 |
| B + A2 | B A2 | B A2 | B | A2 | B | A2 | B | A2 | B | A2 | B | A2 | B | A2 | B | A2 |
| Fountoulaki, 2005 | 20 + 20 | 6 + 7 | 54 ± 6.7 | 56 ± 4.4 | 29.85 ± 5.15 | 30.66 ± 4.22 | 98.1 ± 15.7 | 97.4 ± 12.9 | 155.7 ± 6.8 | 153.0 ± 4.8 | 98.5 ± 4.1 | 97.5 ± 2.1 | 71.8 ± 7.2 | 71.7 ± 7.2 | NR | NR |

Table appendix2( D + A1 ) : The clinical and baseline characteristics of patients

| Study | Sample | Men | Age | | BMI | | LVMI | | SBP | | DBP | | HR | | PWT | |
| --- | --- | --- | --- | --- | --- | --- | --- | --- | --- | --- | --- | --- | --- | --- | --- | --- |
| D + A1 | D A1 | D A1 | D | A1 | D | A1 | D | A1 | D | A1 | D | A1 | D | A1 | D | A1 |
| Gosse, 2000 | 130 + 139 | 121 + 111 | 54.8 ± 10.9 | 54.2 ± 11.3 | 26.7 ± 3.3 | 26.8 ± 3.0 | 142.6 ± 38.6 | 137.6 ± 38.6 | 171.2 ± 10.3 | 170.0 ± 8.8 | 100.5 ± 5.9 | 101.0 ± 6.5 | 69.9 ± 8.6 | 69.1 ± 8.7 | 10.23 ± 1.7 | 10.07 ± 1.66 |
| Roman, 1998 | 28 + 22 | 24 + 13 | 50.1 ± 7.7 | 52.7 ± 6.9 | NR | NR | 92.7 ± 19.9 | 103.6 ± 20.3 | 146 ± NR | 153 ± NR | 93 ± NR | 96 ± NR | NR | NR | NR | NR |
| Senior, 1993 | 9 + 9 | 6 + 6 | 46.8 ± 2.5 | 49.0 ± 3.1 | NR | NR | 151.11 ± 6.26 | 142 ± 6.72 | 178.5 ± 6.5 | 172.3 ± 7.9 | 109.5 ± 2.4 | 106.4 ± 2.1 | NR | NR | NR | NR |
| Dahlof, 1992 | 11 + 12 | NR | 44 ± 3.5 | 48 ± 2.4 | 27 ± 0.9 | 27 ± 0.7 | 121.1 ± 9.8 | 128.9 ± 8.7 | 155.4 ± 5.0 | 155.0 ± 4.0 | 100.5 ± 1.6 | 103.5 ± 1.4 | NR | NR | 11.6 ± 0.6 | 12 ± 0.4 |
| Sihm, 2000 | 14 + 12 | NR | 48 ± 7 | 50 ± 8 | NR | NR | 153 ± 39 | 152 ± 26 | 153 ± 11 | 155 ± 16 | 103 ± 8 | 101 ± 3 | 74 ± 7 | 72 ± 12 | NR | NR |

Table appendix2( D + C ): The clinical and baseline characteristics of patients

| Study | Sample | Men | Age | | BMI | | LVMI | | SBP | | DBP | | HR | | PWT | |
| --- | --- | --- | --- | --- | --- | --- | --- | --- | --- | --- | --- | --- | --- | --- | --- | --- |
| D + C | D C | D C | D | C | D | C | D | C | D | C | D | C | D | C | D | C |
| Okura, 2013 | 21 + 20 | 19 ± 18 | 61.4 ± 10.7 | 62.5 ± 11.2 | 26.1 ± 4.1 | 24.5 ± 4.0 | 137.3 ± 33.6 | 145.8 ± 44.0 | 156 ± 12 | 160 ± 13 | 90.4 ± 8.7 | 90.6 ± 11.2 | 72.3 ± 8.6 | 75.6 ± 13.6 | 10.9 ± 1.9 | 11.0 ± 1.8 |
| Rakic, 2002 | 32 + 17 | 16 + 9 | 51.03 + 5.55 | 52.6 ± 5.33 | NR | NR | 150.72 + 29.99 | 147.6 ± 32.29 | 185.97 + 10.17 | 180.0 ± 9.11 | 107.02 + 3.58 | 106.8 ± 3.25 | 74.16 + 7.01 | 70.1 + 4.87 | NR | NR |
| Sihm, 2000 | 14 + 12 | NR | 48 ± 7 | 47 ± 8 | NR | NR | 153 ± 39 | 182 ± 52 | 153 ± 11 | 168 ± 17 | 103 ± 8 | 108 ± 7 | 74 ± 7 | 72 ± 9 | NR | NR |
| Dey, 1996 | 18 + 18 | 16 ± 15 | 66 ± 5 | 65 ± 5 | NR | NR | 139 ± 37 | 145 ± 35 | 158 ± 16 | 154 ± 10 | 101 ± 6 | 100 ± 4 | 75 ± 11 | 76 ± 9 | NR | NR |
| Senior, 1993 | 22 + 19 | 8 ± 8 | 61 ± 1.6 | 59.9 ± 2.2 | NR | NR | 144.10 ± 5.3 | 170.4 ± 6.6 | 172.4 ± 2.7 | 167.9 ± 2.7 | 104.8 ± 2.0 | 103.1 ± 1.2 | NR | NR | NR | NR |
| Trenkwalder,1994 | 21 + 21 | 6 ± 7 | 79 ± 6 | | NR | NR | 134 ± 21 | 138 ± 25 | 164 ± 15 | 161 ± 18 | 87 ± 8 | 88 ± 11 | 75 ± 14 | 75 ± 10 | NR | NR |
| Giles, 1987 | 8 + 7 | 9 ± 9 | NR | NR | NR | NR | 156 ± 98 | 155 ± 35 | 162 ± 15 | 161 ± 29 | 105 ± 6 | 102 ± 4 | 71 ± 9 | 73 ± 10 | NR | NR |
| Mace, 1985 | 8 + 9 | 4 ± 7 | 40 ± 11 | 40 ± 7 | NR | NR | 107 ± 21 | 126 ± 31 | 164 ± 16 | 173 ± 14 | 97 ± 10 | 109 ± 7 | 76 ± 13 | 77 ± 13 | NR | NR |

Table appendix2( D + A2 and C + A2 ): The clinical and baseline characteristics of patients

| Study  D + A2 | Sample | Men | Age | | BMI | | LVMI | | SBP | | DBP | | HR | | PWT | |
| --- | --- | --- | --- | --- | --- | --- | --- | --- | --- | --- | --- | --- | --- | --- | --- | --- |
|  | D A2 | D A2 | D | A | D | A | D | A | D | A | D | A | D | A | D | A |
| Galzerano,2004 | 25 + 40 | 15 ± 23 | 53 ± 7 | 55 ± 8 | NR | NR | 139 ± 20 | 141 ± 16 | 154 ± 10 | 157 ± 8 | 95 ± 7 | 96 ± 6 | 72 ± 4 | 74 ± 3 | NR | NR |
| Tedesco, 1998 | 28 + 42 | 18 ± 23 | 56 ± 7 | 54 ± 9 | NR | NR | 140 ± 23 | 139 ± 19 | 158 ± 10 | 157 ± 9 | 97 ± 7 | 96 ± 6 | 74 ± 4 | 75 ± 3 | 10.9 ± 1.3 | 10.6 ± 0.9 |
| C + A2 | C A2 | C A2 | C | A2 | C | A2 | C | A2 | C | A2 | C | A2 | C | A2 | C | A2 |
| Yamamoto,2011 | 28 + 29 | 21 ± 24 | 61 ± 9 | 61 ± 13 | NR | NR | 143 ± 47 | 131 ± 33 | 157 ± 18 | 150 ± 21 | 96 ± 14 | 91 ± 12 | 75 ± 13 | 76 ± 10 | NR | NR |
| Yasunari,2004 | 50 + 50 | 31 ± 31 | 64 ± 12 | 62 ± 11 | 24.3 ± 2.8 | 24.1 ± 3.8 | 161 ± 39 | 166 ± 29 | 152 ± 6 | 152 ± 8 | 92 ± 6 | 93 ± 5 | NR | NR | NR | NR |
| Gaudio, 2003 | 30 + 30 | 17 ± 18 | 53.4 ± 13.7 | 50.5 ± 12.7 | NR | NR | 135.6 ± 16.9 | 140.87 ± 13.7 | 168.1 ± 13.0 | 167.8 ± 10.6 | 108.3 ± 5.4 | 107 ± 5.8 | 75.4 ± 8.5 | 77.2 ± 9 | 11.8 ± 0.9 | 11.8 ± 0.6 |

Table appendix3: The results of meta-analysis after sensitive analysis (excluded the study conducted by Gosse)

| A1 |  |  |  |  |
| --- | --- | --- | --- | --- |
| -3.86 ( -9.36 , 2.24 ) | A2 |  |  |  |
| -6.87 ( -14.54 , 1.28 ) | -2.90 ( -11.78 , 6.09 ) | B |  |  |
| 4.34 ( 0.37 , 8.30 )* | 8.22 ( 2.01 , 14.07 )* | 11.22 ( 2.27 , 19.61 )* | C |  |
| 7.65 ( 2.15 , 13.64 )* | 11.56 ( 4.56 , 18.38 )* | 14.50 ( 5.11 , 24.07 )* | 3.34 ( -2.21 , 9.22 ) | D |

A1 = ACEI; A2 = ARB; B = fat-soluble and selective β1-receptor blockers; C = Calcium channel blocker; D = Diuretic.

Table appendix 4: Regression analysis

|  | P value |
| --- | --- |
| Country | 0.466 |
| Sample size | 0.695 |
| Published time | 0.632 |
| Double-dose | 0.519 |
| Duration | 0.921 |
| Monotherapy | 0.913 |

Country: Western country or not; Sample size: n <100 or not; Published time: published before the year of 2000 or not; Doubledose: when blood pressure is controlled unsatisfactorily, double the dose or not; Duration: the period of medication < 1 year or not; Monotherapy: when blood pressure is controlled unsatisfactorily, add other drugs or not.

**References(appendix):**

[1]. N. ÇAĞLAR, I.D., Comparison between nebivolol and ramipril in patients with hypertension and left ventricular hypertrophy_ a randomized open blinded end-point (PROBE) trial. European Review for Medical and Pharmacological Sciences, 2011.

[2]. FOUNTOULAKI, K., et al., Left ventricular mass and mechanics in mild-to-moderate hypertension: Effect of nebivolol versus telmisartan. American Journal of Hypertension, 2005. 18(2): p. 171-177.

[3]. KARIN MALMQVIST, T.K.H.I., Regression of Left Ventricular Mass with Captopril and Metoprolol, and the Effects on Glucose and Lipid Metabolism. BLOOD PRESSURE, 2001.

[4]. Gosse P, R.R.H.G., beta-Blockers vs angiotensinconverting enzyme inhibitors in hypertension: effects on left ventricular hypertrophy. J Cardiovasc Pharmacol, 1990.

[5]. Anan, F., et al., Effects of valsartan and perindopril combination therapy on left ventricular hypertrophy and aortic arterial stiffness in patients with essential hypertension., in Eur J Clin Pharmacol. 2005 Jul;61(5–6):353–9. Epub 2005 May 26. 2005. p. 353–9.

[6]. Uribe Flores JD, H.N.J.C., Losartan versus enalapril in the reduction of left ventricular hypertrophy secondary to systemic arterial hypertension. Arch Cardiol Mex., 2004.

[7]. Cuspidi, C., et al., Comparative effects of candesartan and enalapril on left ventricular hypertrophy in patients with essential hypertension: the candesartan assessment in the treatment of cardiac hypertrophy (CATCH) study., in J Hypertens. 2002 Nov;20(11):2293–300. 2002. p. 2293-300.

[8]. Sanem Nalbantgil, H.Y.C.G., Effects of Valsartan and Enalapril on Regression of Left Ventricular Hypertrophy in Patients with Mild to Moderate Hypertension: A Randomized, Double-Blind Study. Cardiology Department, 2000.

[9]. Bilge, A.K., et al., Effects of amlodipine and fosinopril on heart rate variability and left ventricular mass in mild-to-moderate essential hypertension., in Int J Clin Pract. 2005 Mar;59(3):306–10. 2005. p. 306-10.

[10]. Sabharwal, N.K., et al., Effect of imidapril and nifedipine on left ventricular hypertrophy in untreated hypertension. Clinical drug investigation, 2005. 25(6): p. 367-75.

[11]. Koldas, L., F. Ayan and B. Ikitimur, Short-term effects of rilmenidine on left ventricular hypertrophy and systolic and diastolic function in patients with essential hypertension: comparison with an angiotensin converting enzyme inhibitor and a calcium antagonist., in Jpn Heart J. 2003 Sep;44(5):693–704. 2003. p. 693-704.

[12]. Devereux, R.B., et al., Effects of once-daily angiotensin-converting enzyme inhibition and calcium channel blockade-based antihypertensive treatment regimens on left ventricular hypertrophy and diastolic filling in hypertension: the prospective randomized enalapril study evaluating regression of ventricular enlargement (preserve) trial., in Circulation. 2001 Sep 11;104(11):1248–54. 2001. p. 1248-54.

[13]. Terpstra, W.F., et al., Long-term effects of amlodipine and lisinopril on left ventricular mass and diastolic function in elderly, previously untreated hypertensive patients: the ELVERA trial., in J Hypertens. 2001 Feb;19(2):303–9. 2001. p. 303–9.

[14]. Beltman, F.W., et al., Effects of amlodipine and lisinopril on left ventricular mass and diastolic function in previously untreated patients with mild to moderate diastolic hypertension., in Blood Press. 1998 May;7(2):109–17. 1998. p. 109-17.

[15]. Manolis, A.J., et al., Comparison of spirapril, isradipine, or combination in hypertensive patients with left ventricular hypertrophy: effects on LVH regression and arrhythmogenic propensity., in Am J Hypertens. 1998 Jun;11(6 Pt 1):640–8. 1998. p. 640–8.

[16]. Gaudio, C., et al., Benazepril causes in hypertension a greater reduction in left ventricular mass than does nitrendipine: a randomized study using magnetic resonance imaging., in J Cardiovasc Pharmacol. 1998 Nov;32(5):760–8. 1998. p. 760–8.

[17]. Lombardo, M., et al., Long-term effects of angiotensin-converting enzyme inhibitors and calcium antagonists on the right and left ventricles in essential hypertension., in Am Heart J. 1997 Sep;134(3):557–64. 1997. p. 557-64.

[18]. Sumimoto, T., et al., Both a calcium antagonist and ACE inhibitor reverse hypertrophy in hypertension but a calcium antagonist also depresses contractility., in Cardiovasc Drugs Ther. 1997 Mar;11(1):27–32. 1997. p. 27-32.

[19]. Parodi, O., et al., Comparative effects of enalapril and verapamil on myocardial blood flow in systemic hypertension., in Circulation. 1997 Aug 5;96(3):864–73. 1997. p. 864-73.

[20]. Shimamoto, H. and Y. Shimamoto, Lisinopril reverses left ventricular hypertrophy through improved aortic compliance., in Hypertension. 1996 Sep;28(3):457–63. 1996. p. 457-63.

[21]. Grandi, A.M., et al., Ambulatory blood pressure and left ventricular changes during antihypertensive treatment: perindopril versus isradipine., in J Cardiovasc Pharmacol. 1995 Nov;26(5):737–41. 1995. p. 737-41.

[22]. Kirpizidis, H.G. and G.S. Papazachariou, Comparative effects of fosinopril and nifedipine on regression of left ventricular hypertrophy in hypertensive patients: a double-blind study., in Cardiovasc Drugs Ther. 1995 Feb;9(1):141–3. 1995. p. 141–3.

[23]. van Leeuwen, J.T., et al., Comparative effects of diltiazem and lisinopril on left ventricular structure and filling in mild-to-moderate hypertension., in J Cardiovasc Pharmacol. 1995 Dec;26(6):983–9. 1995. p. 983–9.

[24]. Schulte, K.L., et al., Relation of regression of left ventricular hypertrophy to changes in ambulatory blood pressure after long-term therapy with perindopril versus nifedipine., in Am J Cardiol. 1992 Aug 15;70(4):468–73. 1992. p. 468-73.

[25]. Wang, L.S. and M.Y. Bai, Comparison of the effects of nitrendipine and captopril on the regression of hypertensive left ventricular hypertrophy., in Chin Med J (Engl). 1991 Aug;104(8):645–8. 1991. p. 645–8.

[26]. Kazuhiro Yamamoto, H.O.K.T., The Effect of Losartan and Amlodipine on Left Ventricular Diastolic Function and Atherosclerosis in Japanese Patients with Mild-to-Moderate Hypertension (J-ELAN) study. Hypertension Research, 2011.

[27]. Yasunari, K., et al., Comparative effects of valsartan versus amlodipine on left ventricular mass and reactive oxygen species formation by monocytes in hypertensive patients with left ventricular hypertrophy., in J Am Coll Cardiol. 2004 Jun 2;43(11):2116–23. 2004. p. 2116-23.

[28]. Gaudio, C., et al., Comparative effects of irbesartan versus amlodipine on left ventricular mass index in hypertensive patients with left ventricular hypertrophy., in J Cardiovasc Pharmacol. 2003 Nov;42(5):622–8. 2003. p. 622–8.

[29]. Gosse, P., et al., Regression of left ventricular hypertrophy in hypertensive patients treated with indapamide SR 1.5░mg versus enalapril 20░mg: the LIVE study., in J Hypertens. 2000 Oct;18(10):1465–75. 2000. p. 1465-75.

[30]. Roman, M.J., et al., Differential effects of angiotensin converting enzyme inhibition and diuretic therapy on reductions in ambulatory blood pressure, left ventricular mass, and vascular hypertrophy., in Am J Hypertens. 1998 Apr;11(4 Pt 1):387–96. 1998. p. 387-96.

[31]. Dahlof, B. and L. Hansson, Regression of left ventricular hypertrophy in previously untreated essential hypertension: different effects of enalapril and hydrochlorothiazide., in J Hypertens. 1992 Dec;10(12):1513–24. 1992. p. 1513-24.

[32]. Galzerano, D., et al., Freehand three-dimensional echocardiographic evaluation of the effect of telmisartan compared with hydrochlorothiazide on left ventricular mass in hypertensive patients with mild-to-moderate hypertension: a multicentre study., in J Hum Hypertens. 2004 Jan;18(1):53–9. 2004. p. 53–9.

[33]. Tedesco, M.A., et al., Effects of losartan on hypertension and left ventricular mass: a long-term study., in J Hum Hypertens. 1998 Aug;12(8):505–10. 1998. p. 505-10.

[34]. Takafumi Okura, K.M.J.I., Comparison of the Effect of Combination Therapy with an Angiotensin II Receptor Blocker and Either a Low Dose Diuretic or Calcium Channel Blocker on. Clinical and Experimental Hypertension, 2013.

[35]. Rakic, D., et al., Effects of four antihypertensive monotherapies on cardiac mass and function in hypertensive patients with left ventricular hypertrophy: randomized prospective study., in Croat Med J. 2002 Dec;43(6):672–9. 2002. p. 672–9.

[36]. Sihm, I., et al., Long-term renal and cardiovascular effects of antihypertensive treatment regimens based upon isradipine, perindopril and thiazide., in Blood Press. 2000;9(6):346–54. 2000. p. 346-54.

[37]. Dey, H.M., et al., Comparison of nifedipine GITS and hydrochlorothiazide in the management of elderly patients with stage I-III diastolic hypertension., in Am J Hypertens. 1996 Jun;9(6):598–606. 1996. p. 598-606.

[38]. Trenkwalder, P., et al., Antihypertensive treatment with felodipine but not with a diuretic reduces episodes of myocardial ischaemia in elderly patients with hypertension., in Eur Heart J. 1994 Dec;15(12):1673–80. 1994. p. 1673-80.

[39]. Senior, R., et al., Indapamide reduces hypertensive left ventricular hypertrophy: an international multicenter study., in J Cardiovasc Pharmacol. 1993;22 Suppl 6:S106–10. 1993. p. S106-10.

[40]. Giles, T.D., et al., Comparison of nitrendipine and hydrochlorothiazide for systemic hypertension., in Am J Cardiol. 1987 Jul 1;60(1):103–6. 1987. p. 103–6.

[41]. Mace, P.J., et al., Regression of left ventricular hypertrophy in hypertension: comparative effects of three different drugs., in J Cardiovasc Pharmacol. 1985;7 Suppl 2:S52–5. 1985. p. S52–5.
